# Pseudobulbar Affect Presenting as Hypomania

**DOI:** 10.7759/cureus.7308

**Published:** 2020-03-18

**Authors:** Eduardo D Espiridion, Cassandra Mitchell, Shreeja Kadakia

**Affiliations:** 1 Psychiatry, Reading Hospital Tower Health, West Reading, USA; 2 Psychiatry, Drexel University College of Medicine, Philadelphia, USA; 3 Psychiatry, West Virginia School of Osteopathic Medicine, Lewisburg, USA; 4 Psychiatry, West Virginia University School of Medicine, Martinsburg, USA; 5 Psychiatry, Philadelphia College of Osteopathic Medicine, Philadelphia, USA; 6 Medicine, Drexel University School of Medicine, Philadelphia, USA

**Keywords:** pseudobulbar affect, stroke, hypomania, mania, depression, antipsychotics, antidepressants

## Abstract

Pseudobulbar affect (PBA) is a behavioral syndrome associated with various neurological conditions that typically manifests as uncontrollable laughing or crying. PBA can significantly impact the quality of life of patients affected as these spells can be inappropriate to the social setting or incompatible with the patient's emotional state. The underlying mechanism of PBA appears to be associated with disinhibition in neuronal pathways involving dopamine, serotonin, and glutamate, but the exact mechanism remains unclear. One hypothesis for the pathology of PBA is that it is the result of disruption of the corticopontine-cerebellar circuits, impairing cerebellar modulation of affect, and leading to uncontrolled emotional lability. Stroke, and other neurological disorders, interrupt these neuronal circuits causing disinhibition of the voluntary control of emotional expression. It is extremely important to recognize and appropriately diagnose the condition. We present a case report of an 85-year-old female patient who presented with a thalamic stroke, and she subsequently developed hypomania with symptoms of decreased need for sleep, mood lability, pressured speech, and religious preoccupation. This case discusses a unique presentation of PBA with hypomania.

## Introduction

Pseudobulbar affect (PBA) is an extremely rare symptom that manifests secondary to a variety of disease states as uncontrollable laughing or crying. The outburst of crying or laughing may be different than the patient's current emotional state. It is especially common in patients with Parkinson's disease, multiple sclerosis, and stroke. PBA symptoms were present in about 17.5% of the nursing home residents with neurological condition [1]. As PBA often involves uncontrollable crying, it may be confused with depression. The crying spells are not congruent with the patient's emotional state and they may not able to stop it. Patients are often euthymic in between episodes and often become overly preoccupied with thoughts of these episodes recurring. As a result, it significantly diminishes patient's quality of life as they are frustrated and anxious about these episodes [2]. One in five stroke survivors experience acute PBA and one in eight survivors experience PBA symptoms after six months [3]. 

## Case presentation

**Figure 1:**
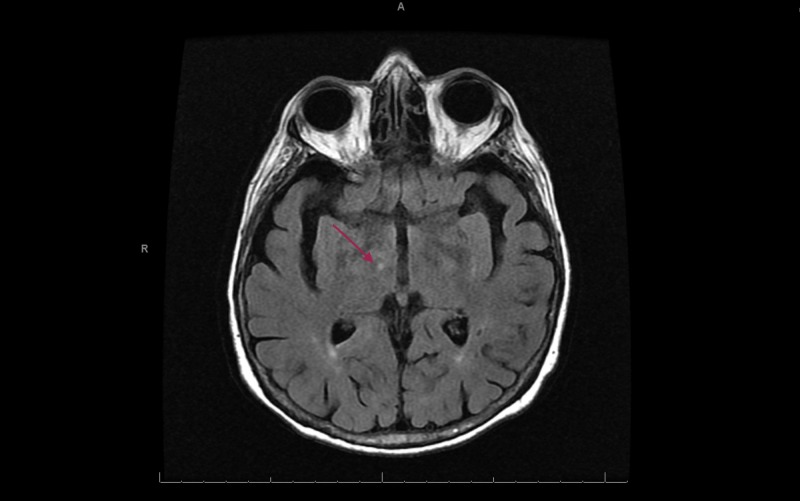
MRI brain axial T2 of acute to subacute right thalamic lacunar infarct.

**Figure 2:**
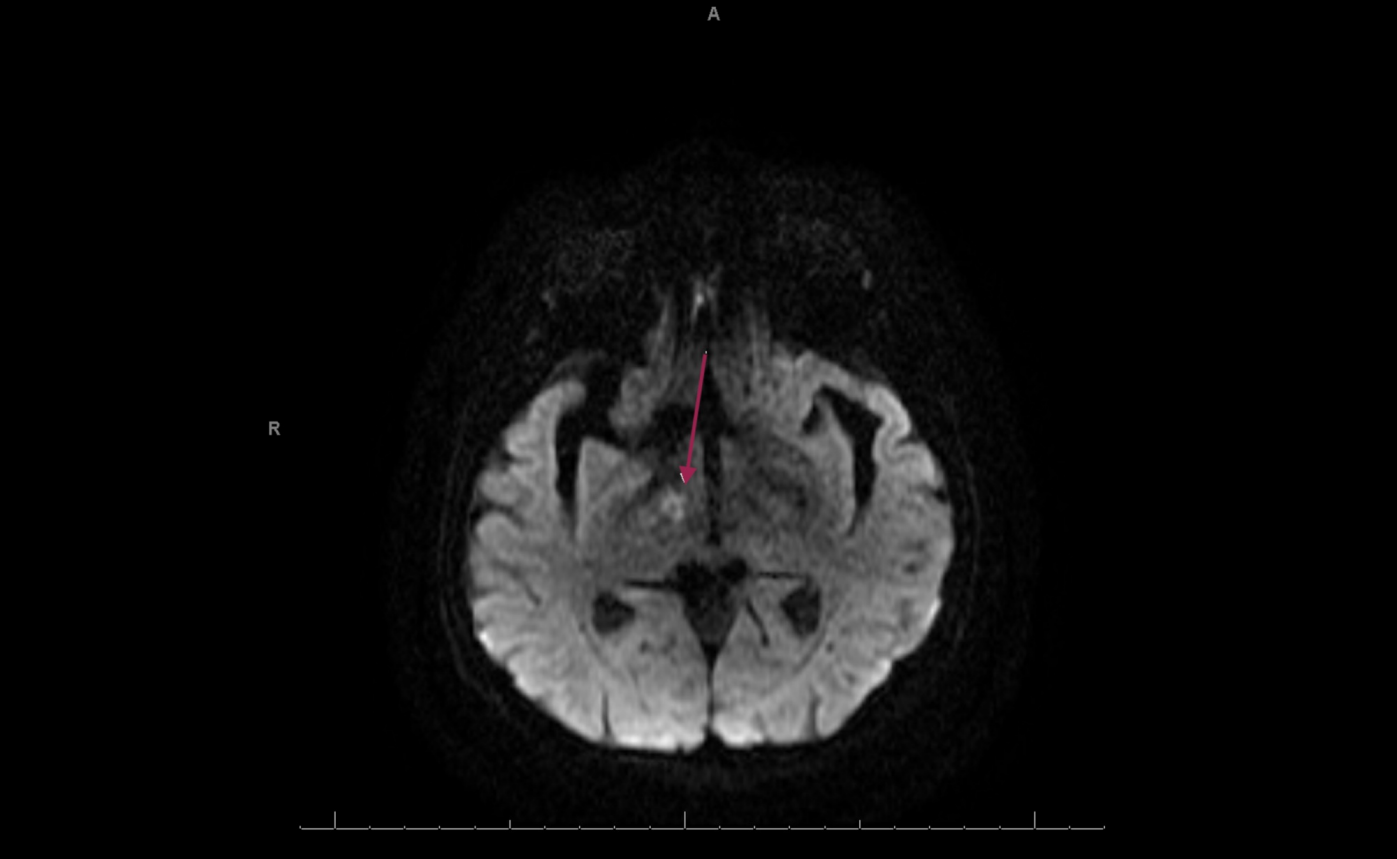
MRI brain axial DWI of acute to subacute right thalamic lacunar infarct. DWI, diffusion weighted imaging

**Figure 3:**
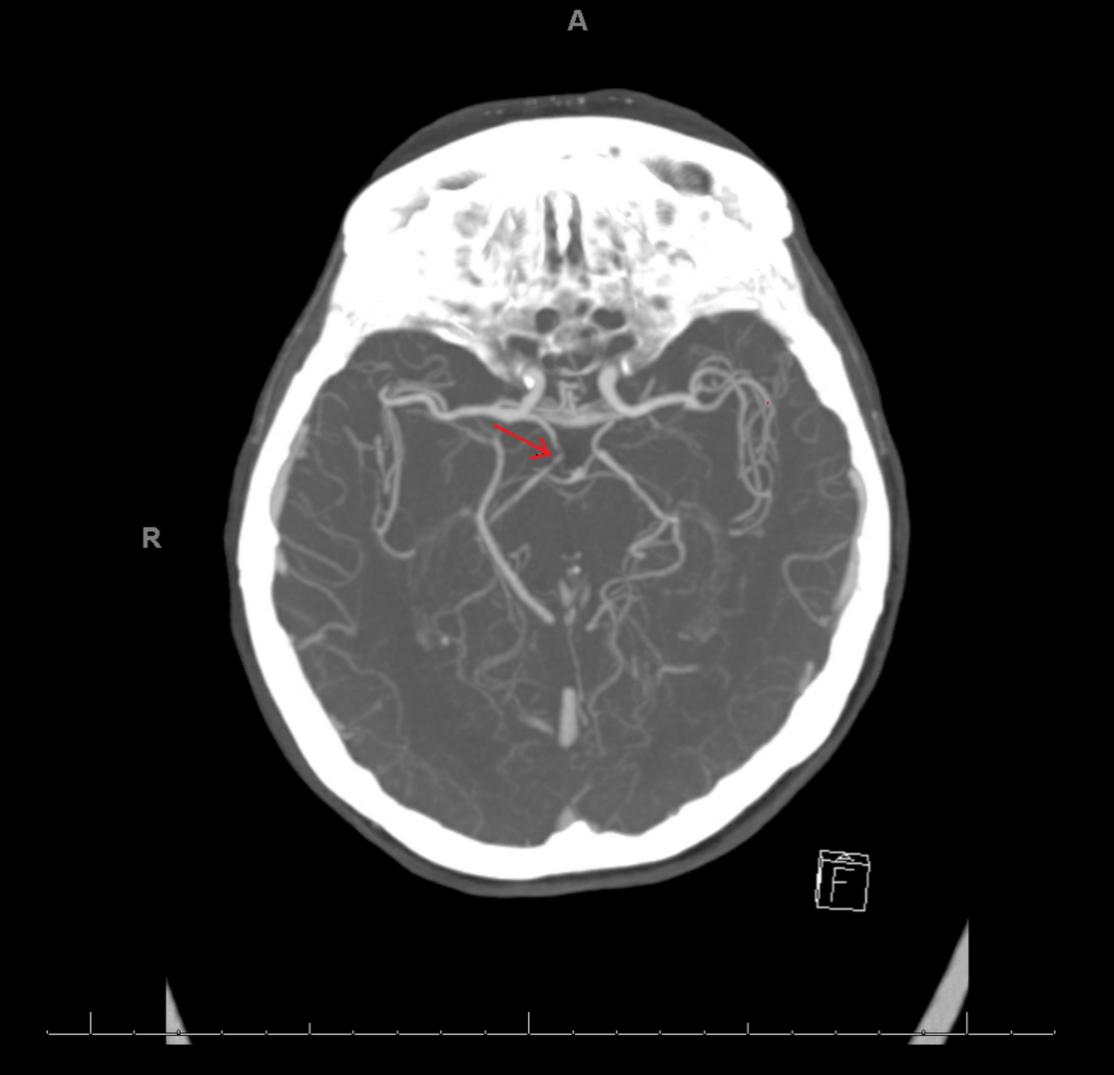
CTA of the head shows a mild to moderate short segment narrowing of the distal right P-comm artery, multi-focal narrowing of the right posterior cerebral artery. CTA, CT angiography

This is a case of an 85-year-old female with a long history of depression, which had been well-controlled with sertraline for 19 years. She reports several episodes of hypomania when she would experience high levels of energy and she would engage in spending sprees. None of these symptoms were evaluated or treated. She describes herself as an energetic individual and her mood as being "high" most of the time. The patient has been on apixaban for a deep vein thrombosis, but it was discontinued several months prior to presentation. She denied any history of atrial fibrillation. The patient presented to the ED of a local community hospital after she was observed to be confused. Her husband also noticed a left-sided facial droop and severe left-sided weakness. This happened approximately one hour after waking up from a nap. Her symptoms markedly improved while on their way to the hospital. A tissue plasminogen activator (TPA) was deferred as the National Institute of health Stroke Scale was zero. Subsequent MRI of the brain without contrast was significant for acute to subacute thalamic lacunar infarct (Figures 1 and 2). A CT angiography (CTA) of the head and neck was significant for markedly attenuated mid and distal right P1 segment with patent right posterior communicating artery, and multi-focal narrowing of the right posterior cerebral artery (Figure 3). 

A psychiatric consult was requested because the patient presented with hypomania with psychomotor agitation. She presented with mood lability, pressured speech, and distractibility. She was religiously preoccupied, and she reported "an encounter with the Lord." She slept for three hours a night before she went to the ER. She admitted to a long psychiatric history of depression with sad mood, crying spells, low energy level, sleep disturbances, poor concentration, and feelings of hopelessness. She denied any suicidal ideations. There were no delusions or hallucinations at the time of the evaluation. She was not physically aggressive or agitated. The patient reported that her mother had "manic-depression" with multiple suicide attempts, her brother had schizophrenia, and her great-uncle was in a long-term state psychiatric facility, and her daughter was diagnosed with depression. The patient was started on lamotrigine at 25 mg per day and sertraline was discontinued. It was thought that the Zoloft was contributing to the patient's hypomania. She was discharged home the next day with outpatient follow-up. Five days after her discharge to home, the patient was returned to the local ER of this community hospital with manic symptoms of euphoria, racing thoughts, pressured speech, verbal aggression, impaired judgement, and decreased need for sleep. These symptoms worsened in spite of her compliance with her lamotrigine. She was admitted in the behavioral health unit and started on risperidone 0.5 mg at bedtime. Her lamotrigine dose was increased to 50 mg per day. The patient's symptoms improved after a few days and she was referred to the outpatient psychiatrist. 

## Discussion

Pseudobulbar affect is a condition associated with multiple neuropsychiatric pathologies, including stroke. It is often missed as a diagnosis and often mistaken as clinical depression or bipolar disorder. Early identification and initiation of treatment are especially important for patients who experience PBA secondary to strokes. PBA screening consists of the Center for Neurologic Study-Liability Scale (CNS-LS) which comprises seven questions with each question getting a score from 1 to 5, with a total of 35 points. The PLCS has 18 questions with a score range of 0-3 for each question [2]. 

Current research suggests that PBA is caused by disruptions in the usual mechanisms of dopamine, serotonin, and glutamate in emotional expression. Imaging studies have revealed that there is a reduced serotonin and dopamine transmission and increased glutamate transmission. The treatment involves increasing serotonin and dopamine transmission and reducing glutamate stimulation [4]. Clinical trials have shown that selective serotonin reuptake inhibitors and tricyclic antidepressants have been efficacious in treating PBA. Nortriptyline, citalopram, and imipramine were especially effective in stroke patients [5]. Dextromethorphan, a glutamate antagonist, may also be used to inhibit glutamate activity at the N-methyl-D-aspartate (NMDA) receptor as well as sigma-1 receptor [5]. Antidepressant therapies have been used in the past. A medicine combination that contains dextromethorphan hydrobromide and quinine sulfate is approved by the United States Federal Drug Administration (FDA) for PBA. 

Strokes interrupt circuits projecting to the cerebellum and brainstem, causing disinhibition of voluntary control of emotional expression [6]. Emotional dysregulation may present as depressive or manic symptoms. In our case presentation, the patient presented with manic symptoms instead of depressive symptoms. She presented with symptoms of mood lability, pressured speech, psychomotor agitation, and racing thoughts. It is extremely important to diagnose PBA even if it presents as mania. The CNS-LS has so far proven to be the most accurate in terms of diagnosis of PBA in stroke patients. Data from the PBA Registry Series (PRISM) demonstrated that higher CNS-LS scores were associated with greater use of antipsychotic and antidepressant medications [6]. PBA is characterized by the pathological crying spells and laughter, emotional incontinence, and mood lability. This patient presented with laughter that was disproportionate to the social context as well as her emotional experience. The use of the Diagnostic and Statistical Manual of Mental Disorders Fifth Edition and the performance of a structured interview may minimize confusion between PBA and bipolar disorder. PBA is a neurological impairment and not considered a mental illness.

## Conclusions

Stroke-induced disturbances in neuronal circuits may cause malfunctioning of the dopamine, serotonin, and glutamate pathways which present as behavioral disinhibition. Patients may have spells of crying or laughing which are not consistent with their emotional state and cause significant impairment of their quality of life, mental, and physical health. Data from PRISM show that the CNS-LS scale is currently best at diagnosing PBA in stroke patients. Our case discussed a unique presentation of PBA in which the patient had symptoms of hypomania instead of depression. Her condition improved after the inpatient psychiatric hospitalization and treatment with antipsychotics.
